# An individual alginate lyase is effective in the disruption of *Laminaria digitata* recalcitrant cell wall

**DOI:** 10.1038/s41598-021-89278-1

**Published:** 2021-05-06

**Authors:** Mónica Costa, Luís Pio, Pedro Bule, Vânia Cardoso, Cristina M. Alfaia, Diogo Coelho, Joana Brás, Carlos M. G. A. Fontes, José A. M. Prates

**Affiliations:** 1 CIISA - Centro de Investigação Interdisciplinar Em Sanidade Animal, Faculdade de Medicina Veterinária, Universidade de Lisboa, 1300-477, Lisboa, Portugal; 2 NZYTech - Genes and Enzymes, Estrada do Paço Do Lumiar, Campus do Lumiar, Edifício E, 1649-038 Lisboa, Portugal

**Keywords:** Carbohydrates, Enzymes

## Abstract

In the present study, 199 pre-selected Carbohydrate-Active enZymes (CAZymes) and sulfatases were assessed, either alone or in combination, to evaluate their capacity to disrupt *Laminaria digitata* cell wall, with the consequent release of interesting nutritional compounds. A previously characterized individual alginate lyase, belonging to the family 7 of polysaccharide lyases (PL7) and produced by *Saccharophagus degradans*, was shown to be the most efficient in the in vitro degradation of *L. digitata* cell wall. The alginate lyase treatment, compared to the control, released up to 7.11 g/L of reducing sugars (*p* < 0.001) and 8.59 mmol/100 g dried alga of monosaccharides (*p* < 0.001), and reduced cell wall fluorescence intensity by 39.1% after staining with Calcofluor White (*p* = 0.001). The hydrolysis of gel-forming polymer alginate by the alginate lyase treatment could prevent the trapping of fatty acids and release beneficial monounsaturated fatty acids, particularly 18:1*c*9 (*p* < 0.001), to the extracellular medium. However, no liberation of proteins (*p* > 0.170) or pigments (*p* > 0.070) was observed. Overall, these results show the ability of an individual alginate lyase, from PL7 family, to partially degrade *L. digitata* cell wall under physiological conditions. Therefore, this CAZyme can potentially improve the bioavailability of *L. digitata* bioactive compounds for monogastric diets, with further application in feed industry.

## Introduction

Due to an increasing interest in the use of macroalgae for food and feedstuffs^1^, as well as for pharmaceutical industries, organic fertilizers, eutrophication inhibition, bioremediation and biogas generation^1^, their cultivation has been steadily growing over the last decade. The nutritional profile of macroalgae, although variable among species and depending on growth location and harvesting season^1^, consists of numerous vitamins, minerals, pigments, phenolic compounds, carbohydrates and high quality proteins^1^. Carbohydrates comprise a high proportion of macroalgae biomass (from 4 to 76% dry matter, DM)^2^, whereas lipids are usually found in small amounts (< 5% DM) with values up to 1.13% DM in brown algae^3^. However, their lipid profile can be rich in monounsaturated (MUFA) and polyunsaturated (PUFA) fatty acids^4–6^, which might have beneficial effects on human health^7^.

*Laminaria* sp*.* are seawater multicellular eukaryotic and autotrophic brown macroalgae, which are amongst the most cultivated seaweeds worldwide, representing the largest biomass in coastal regions^8^. Brown macroalgae have a distinct carbohydrate-rich cell-wall^9,10^, which comprises up to 45% DM of alginate and fucose-containing sulphated polysaccharides (FCSPs) (fucans or homofucans and fucoidans or heterofucans), as well as small amounts of cellulose (1 to 8% DM)^11^, β-1,3 glucans, unbranched mixed-linkage β-D-glucans (1,3-and-1,4-β-D-glucose residues) masked by alginate^12^ and arabinogalactans linked to proteins^13^. The proportion of alginates, fucoidans and cellulose was found to be 3:1:1^10^. Alginate is a linear polysaccharide composed of only two epimers, β-1,4-D-mannuronic (M) and α-1,4-L-guluronic (G) acids, arranged in heteropolymeric (MG) or homopolymeric (MM or GG) blocks along the polymer chain^11^. FCSPs are composed of two different backbones with α-1,3- or alternating α-1,3-/α-1,4-linked L-fucose residues. These motifs form acetylated or branched structures presenting one to three sulphate esters on positions *O*-2, *O*-3 or *O*-4^11^. Laminarin is a polysaccharide located in intracellular vacuoles that constitutes the carbon storage of macroalgae and is composed of (1,3)-β-D-glucopyranose residues with some 6-O-branching in the main chain and β-1,6-intrachain links^14,15^. These polysaccharides were found to have bioactive properties with relevance for potential applications in functional foods and feeds, cosmetics and pharmaceutical products^13–15^. For instance, alginate was shown to reduce blood pressure and cholesterol and to have antimicrobial and anticancer activities^15^; FCSPs were described as antioxidant, anti-inflammatory, immune-stimulant, antimicrobial and anticancer compounds^13,15^; and laminarin was reported as an anticancer, anti-inflammatory, immunostimulatory, anticoagulant and antioxidant^14,15^ agent.

However, cell wall carbohydrates are organized in a complex cross-linked matrix, resulting in a highly recalcitrant cell wall that resists breakage and serves as a natural defence mechanism for algae^9^. In fact, alginate cross-links with phenolic compounds and constitutes gel-forming and hydroscopic polymers that control cell wall rigidity^11^. These polymers form a network that embed fucose-containing sulphated polysaccharides. The latter are tightly assembled to cellulose microfibrils by cross-linkage^11^. The intricate macroalga cell walls have been described to exert anti-nutritional effects for monogastric animals, by trapping valuable nutrients, with a concomitant decrease in the efficiency of feed digestion and absorption^16^. The presence of complex polysaccharides in seaweed cell walls can also decrease the rate of algae biomass hydrolysis during the production of renewable energies, thus reducing bioethanol and biogas yields^17^.

Mechanical processes, such as hammer mill, are usually applied for incorporation of seaweed in diets for monogastric animals^1^. However, mechanical methods are less specific for macroalgae cell disruption than enzymatic procedures. Therefore, the latter can be more advantageous for the release of algae valuable nutritional and bioactive compounds and increase of their bioavailability^18^. The effectiveness of exogenous enzymes (*i.e.* cellulases, xylanases^19–22^ and a mixture of carbohydrases^18^) on hydrolysing algae biomass with an increase of protein extraction or digestibility was previously demonstrated for green (i.e. *Ulva rigida*)^18^ and red (e.g. *Palmaria palmata*, *Gracilaria* sp. and *Chondrus* sp.)^19–22^ seaweeds. Other studies reported the use of cellulases, alginate lyases^23–25^ and a carbohydrase mixture^26^ for the degradation of brown macroalgae biomass (*Laminaria digitata*^23,25^, *Saccharina latissima*^24,25^ and a mixture of different species including *Sargassum* sp.^26^) envisaging biotechnological applications. These applications consisted in the production of bioethanol and biogas^23,25^, algae saccharification^24^ and extraction of bioactive compounds^26^.

Therefore, exogenous Carbohydrate-Active enzymes (CAZymes) could be a suitable option to deconstruct macroalgae cell wall, similarly to what was recently described by our research team for microalgae^27,28^. Moreover, these exogenous enzymes were shown to improve the nutritional value of cereal-based diets^29,30^, with the consequent industrial application as feed additives for poultry and pigs^31^. Additionally, the use of sulfatases could be of major importance for the degradation of the recalcitrant structure of branched and sulphated FCSPs, as reported in a recent study^32^. Thus, we hypothesised that CAZymes and sulfatases could, individually or in combination, degrade the recalcitrant *L. digitata* cell wall, with the consequent improvement of nutrients bioavailability. Cell wall disruption was assessed by fluorescence microscopy, reducing sugars and oligosaccharides profile after incubation of macroalga with the enzymes. The release of nutritive and bioactive compounds from macroalga, following the enzyme treatment, was assessed by quantifying proteins, pigments and fatty acids.

## Results

### CAZymes and sulfatases selection and evaluation of expression and purity of recombinant enzymes

All of the CAZYmes and sulfatases selected for the initial screen were chosen for their activity, either determined or predicted, towards specific compounds of brown macroalgae cell walls, like alginate, FCSFs and cellulose^11^. Furthermore, most are produced by marine halophilic bacteria, which are organisms likely adapted to feed on algae biomass. The catalytic activity and the biochemical properties of the majority of the candidates were previously described in the literature and their amino acid sequences can be accessed in Genbank (see Supplementary Table S1). Seventeen of the selected CAZYmes (ID 166 to 174, 184, 189 to 195) did not have their activity and substrate specificity previously characterized, but were selected due to sharing high homology (see Supplementary Table S1 for Genbank accession numbers) to some of the other well-characterized candidates.

In order to evaluate the soluble protein yield for each enzyme, a qualitative scale was used based on the protein concentration (g/L): -, 0.0; + , 0.1 > 2.1; +  + , 2.1 > 4.1; +  +  + , 4.1 > 6.1; +  +  +  + , > 6.1 (see Supplementary Table e S1). From the 199 recombinant enzymes, 23 did not express (-), 81 had low expression levels or were mostly insoluble ( +) and 95 had good expression and solubility levels (+ + , +  +  + , +  +  + +). Among low expressing enzymes, 4 were slightly degraded (ID 5, 69, 73 and 129). Among high expressing enzymes, 1 had low solubility, 1 was degraded and 3 (ID 147, 151 and 184) had a different SDS-PAGE migration pattern than what was expected from the calculated molecular weight (data not shown). All soluble protein fractions were enriched by a high throughput IMAC protocol before the activity screens (see Supplementary Fig. S1).

### Screening of individual enzymes for *Laminaria digitata* cell wall disruption

Each one of the 176 CAZymes and sulfatases with low to high expression levels (see Supplementary Table S1) was individually incubated with a macroalga suspension in phosphate buffered saline (PBS) solution for an evaluation of their ability to degrade *L. digitata* cell wall. The majority of enzymes was unable to deconstruct alga biomass (see Supplementary Table S2), but 8 individual enzymes (ID 6, 18, 20, 21, 22, 28, 29 and 46) had a measurable capacity to degrade the cell wall of *L. digitata*, as shown in Table 1. This ability was assessed by both the release of reducing sugars, as evaluated through the 3,5-dinitrosalicylic acid (DNSA) method, and the decrease of fluorescence intensity from Calcofluor white stained cell walls. The fact that brown macroalgae cell walls form an intricate carbohydrate structure^11^ will allow the evaluation of cell wall integrity loss by assessing the reduction of Calcofluor white staining fluorescence intensity, even though the dye only binds specifically to minor compounds of the cell wall (cellulose and, to some extent, mixed-liked 1,3–1,4-β-glucans)^12,33^. Therefore, the data in Table 1 is presented according to two qualitative scales: the first scale is based on the amount of released reducing sugars (g/L): -, 0.00 < 2.77; + , 2.77 < 3.99; +  + , 3.99 < 5.20; +  +  + , 5.20 < 6.42; and +  +  +  + , > 6.42; whereas the second one is based on the decrease of fluorescence intensity (%): -, 0.00 < 9.92; + , 9.92 < 20.0; +  + , 20.0 < 29.9; and +  +  + , 29.9 < 38.5; +  +  +  + , > 38.5. For the enzymes with ID 6 and 46, the release of reducing sugars (average of 0.46 g/L) was in the lower level considered in Table 1 and they caused only a low to intermediate decrease of fluorescence intensity (up to 22.3%). However, enzyme with ID 46 was selected because its predicted substrate is α-linked L-fucopyranosyl units, which are the main residues found in one of the major components of brown seaweed cell walls, the FCSPs^11^. Although the substrate for the enzyme with ID 6 (mixed linked 1,3–1,4-β-glucans) is only a minor component of brown macroalgae cell walls, its insoluble nature can impact the degradation of algae cell wall, mainly through the association with a major cell wall polysaccharide, the alginate^12^.

**Table 1:** Screening of the selected individual CAZymes for *Laminaria digitata* cell wall disruption.

ID	Name	Category	EC number	Main substrate	Released reducing sugars scale	Decreased fluorescence intensity scale
6	Cellulase (Cel73;Cell73)	Cellulases	3.2.1.4	1,3–1,4-β-glucans and soluble 1,4-β-glucans	− (0.26 g/L)	+ (14.6%)
18	Laminarinase A (LamA)	1,3-β-Glucanases	3.2.1.-/3.2.1.39	Laminarin (1,3–1,4/1,6-β-glucans)	+ + (5.16 g/L)	+ + + + (40.1%)
20	Endo-guluronate lyase (AlyA1;zobellia_1182)	Poly-α-guluronate lyases	4.2.2.11	Sodium alginate/β-elimination reaction	+ + + + (6.63 g/L)	+ + + + (49.8%)
21	β-1,3–1,4-glucanase P2 (LicP;GluB)	1,3–1,4-β-Glucanases	3.2.1.73	1,3–1,4-β-glucans	+ + (5.10 g/L)	+ + + + (51.6%)
22	Alginate lyase / poly-β-mannuronate (Sde_2547)	Alginate lyases	4.2.2.3	Alginates and oligoalginates	+ + + + (6.78*-7.11** g/L)	+ + + + (39.1**—46.5* %)
28	Cellobiohydrolase (CbhA; Cthe_0413)	Cellobiohydrolases	3.2.1.-/3.2.1.91	Amorphous and crystalline cellulose	+ + + (5.42 g/L)	+ + + + (57.0%)
29	Lytic transglycosylase A (MltA; Mlt;b2813)	Murein lyase/Exomuramidase	4.2.2.n1	Murein glycan strands and insoluble, high-molecular weight murein sacculi	+ + (4.42 g/L)	+ + + + (42.9%)
46	α-L-fucosidase C(AlfC; LCABL_29340 possible fragment)	Fucosidases	3.2.1.51 F1-6Gn	p-nitrophenyl-α-L-fucopyranoside	− (0.65 g/L)	+ + (22.3%)

Each enzyme is presented with the project identification number (ID), name, category, EC number, main substrate and qualitative scales of reducing sugars and fluorescence intensity. The following qualitative scales were defined: (1) amount of released reducing sugars (g/L): − , 0.00 < 2.77; + , 2.77 < 3.99; +  + , 3.99 < 5.20; +  +  + , 5.20 < 6.42, +  +  +  + , > 6.42; (2) decrease of fluorescence intensity (%): − , 0.00 < 9.92; + , 9.92 < 20.0; +  + , 20.0 < 29.9; +  +  + , 29.9 < 38.5; +  +  +  + , > 38.5. The numeric values of released reducing sugars and decreased fluorescence intensity obtained for alginate lyase (ID 22), the most active enzyme on cell wall disruption, are also presented. *Initial screening, **final incubation.

### Selection of the most active enzymes and assessment of their synergistic action

**Figure 1:**
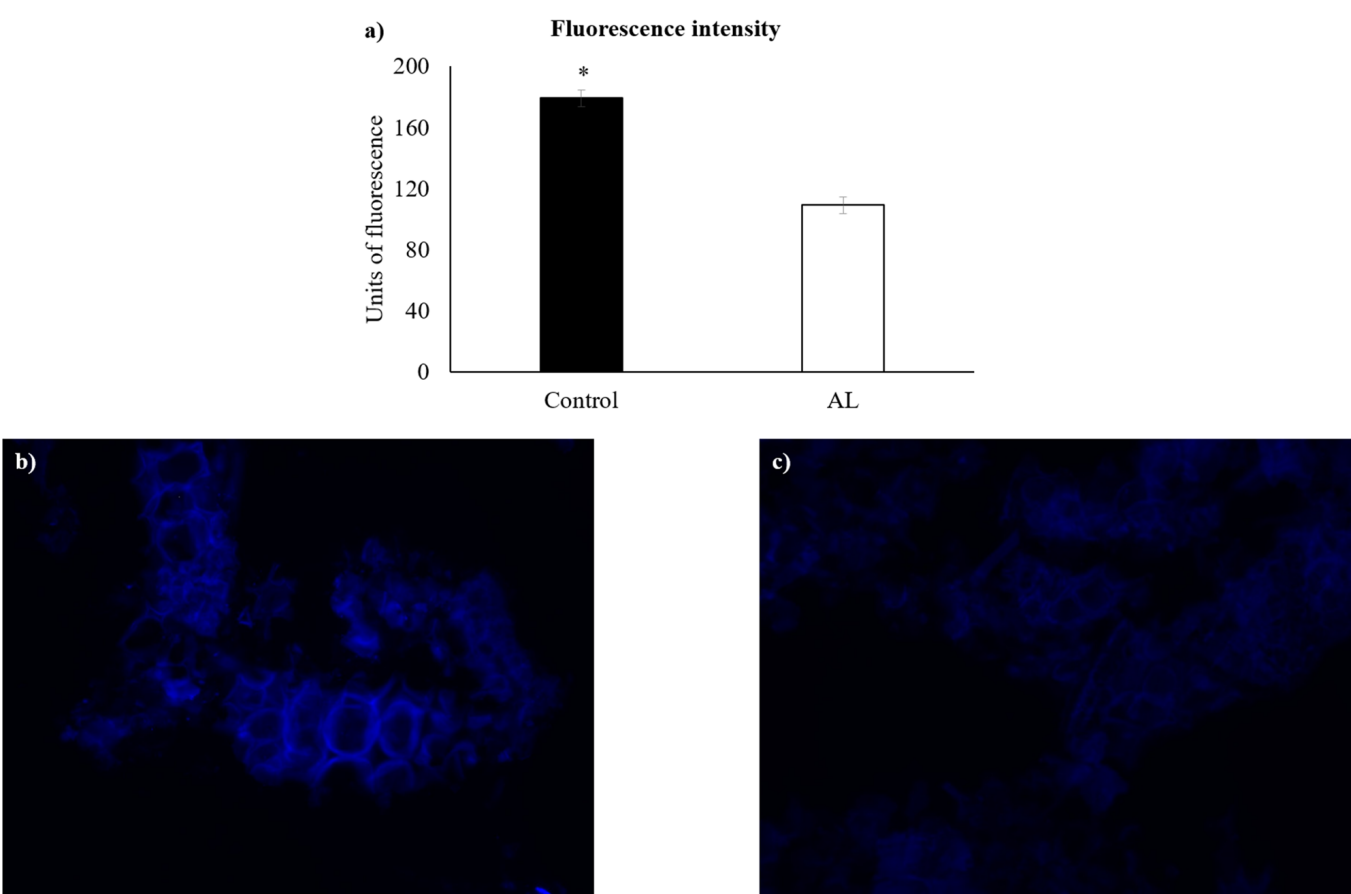
(**a**) Fluorescence intensity derived from Calcofluor White staining for control assay and alginate lyase (AL; ID 22) treatment. Asterisk denotes statistical difference at *p* = 0.001. (**b**) and (**c**): fluorescence images (× 400) of *Laminaria digitata* suspension stained with Calcofluor White for control assay and alginate lyase treatment, respectively.

**Figure 2:**
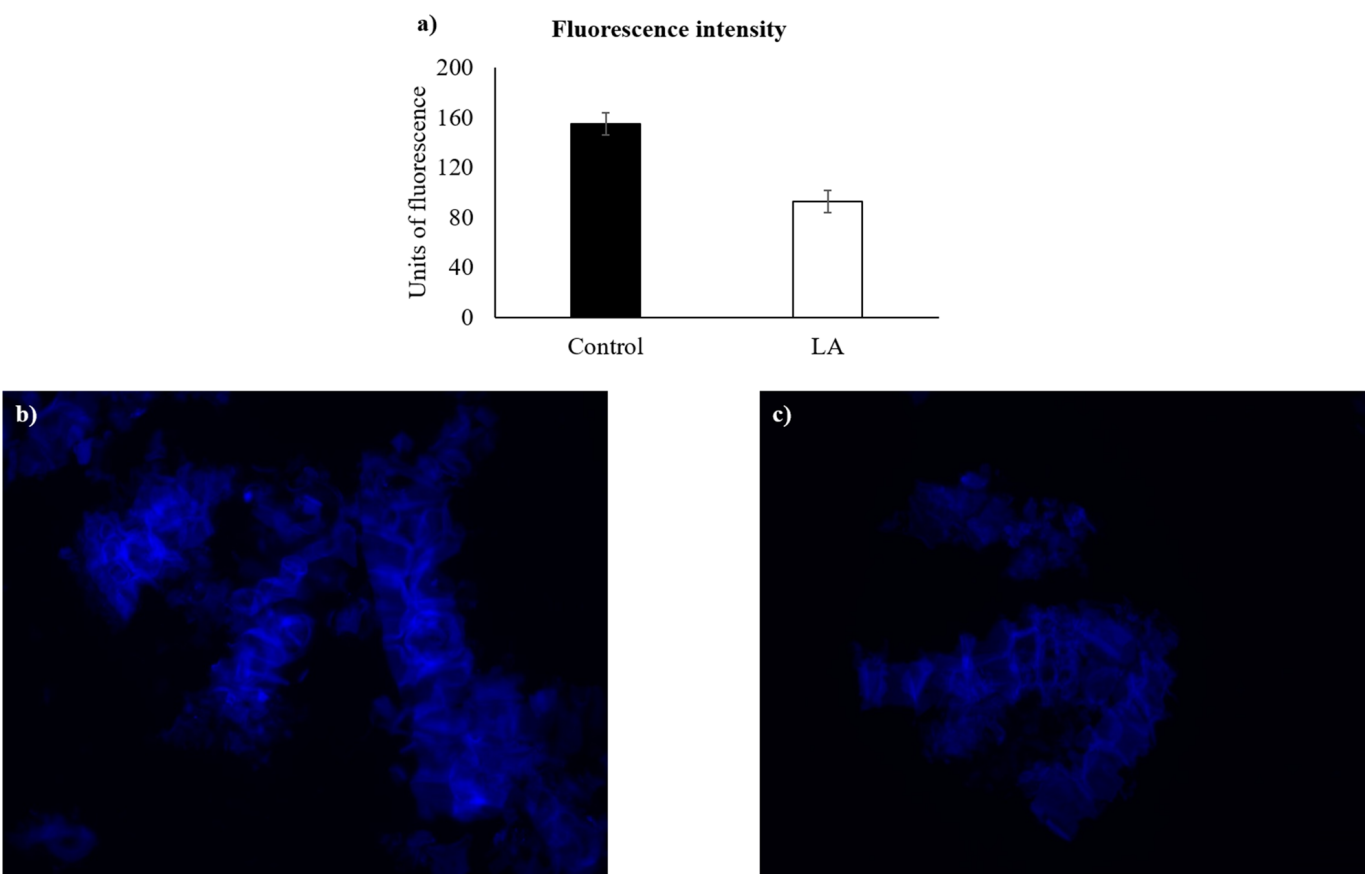
(**a**) Fluorescence intensity derived from Calcofluor White staining for control assay and laminarinase (LA; ID 18) treatment. No statistical differences were observed (*p* = 0.070). (**b**) and (**c**) fluorescence images (× 400) of *Laminaria digitata* suspension stained with Calcofluor White for control assay and laminarinase treatment, respectively.

**Figure 3:**
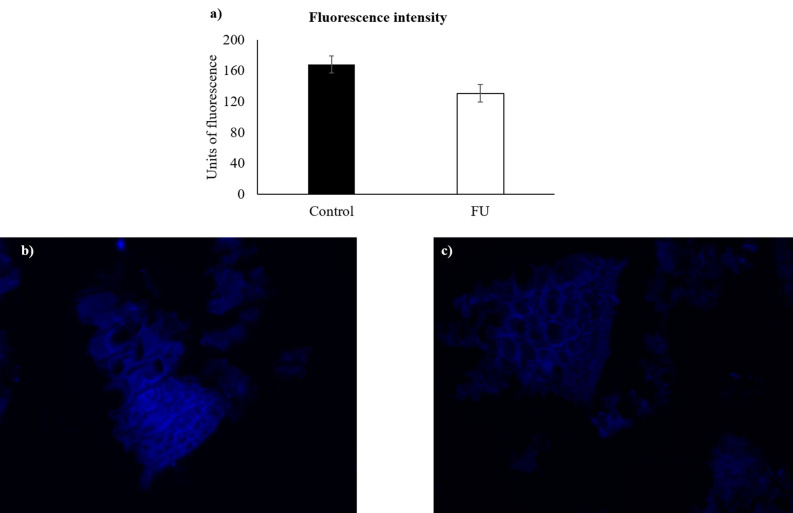
(**a**) Fluorescence intensity derived from Calcofluor White staining for control assay and fucosidase (FU; ID 46) treatment. No statistical differences were observed (*p* = 0.195). (**b**) and (**c**) fluorescence images (× 400) of *Laminaria digitata* suspension stained with Calcofluor White for control assay and fucosidase treatment, respectively.

In order to disclose synergistic actions among individual enzymes, an eight-enzyme mixture based on the initial screening (Table 1), was compared to a three-enzyme mixture (ID 18, 22 and 46) (Table 2, Figs. 1, 2 and 3). These three enzymes were selected based on their organism of origin, thermostability and main substrate. Indeed, laminarinase (ID 18) and alginate lyase (ID 22) were isolated from marine and halophilic bacteria (*Thermotoga napolitana*^34^ and *Saccharophagus degradans*^35^, respectively), and were described as being thermoresistant with optimum catalytic activities at 85 to 95 ºC^34^ and 50 ºC^35^, respectively. Although the enzyme with ID 46 was from a non-marine and non-halophilic bacterium (*Lactobacillus casei*)^36^, it was relatively thermostable, with an optimum temperature of 42 ºC^36^, and acted towards a main constituent of brown algae cell wall (α-linked L-fucopyranosyl units)^11^.

**Table 2:** Evaluation of additive and synergistic effects between the most active CAZymes (project identification number, ID) on the release of reducing sugars from *Laminaria digitata* cell wall.

Enzymes*	Released reducing sugars (g/L)	*p*-value (Mix 8 versus ID 22)	*p*-value (Mix 3 versus ID 22)
Mix 8	6.74	0.001	0.443
Mix 3	6.17		
ID 18	4.57		
ID 22	6.25		
ID 46	0.50		

*Mix 8: ID 6, 18, 20, 21, 22, 28, 29 and 46; Mix 3: ID 18, 22 and 46.

The eight-enzyme mixture led to a release of reducing sugars of 6.74 g/L, which corresponded to an increase of only 0.57 g/L compared to the three-enzyme mixture. Then, the latter mixture was compared to the activities of each enzyme composing it. It was observed that, when enzyme ID 22 enzyme was individually incubated with *L. digitata* suspension, the value of released reducing sugars was identical (p = 0.443) to the three-enzyme mixture (6.17 g/L). In contrast, the released reducing sugars by the other individual enzymes (4.57 g/L for enzyme ID 18, and 0.50 g/L for enzyme ID 46) were significantly lower than that of mixture (p < 0.001). Altogether, these results indicate the absence of significant (p > 0.050) additive or synergistic effects among enzymes.

The ratios of released reducing sugars were found to be: alginate lyase *versus* three-enzyme mixture = 101.3%; alginate lyase *versus* laminarinase = 136.6%, and alginate lyase *versus* fucosidase = 1242%. Regarding the above values, the alginate lyase ID 22 was selected as the most active enzyme for the degradation of *L. digitata* cell wall.

### Effect of alginate lyase on *Laminaria digitata* cell wall integrity

The extension of released reducing sugars and decreased fluorescence pixels of stained cell walls promoted by the selected alginate lyase (ID 22; Provisional Patent number, INPI, Portugal) are presented in Table 1. The latter is also illustrated in Figs. 1a, b and c. The amount of reducing sugars (7.11 g/L) was significantly increased (p < 0.001), whereas the number of pixels (179 to 109; 39.1% decrease of fluorescence intensity) was significantly reduced (p = 0.001) with the enzyme ID 22, when compared to the control assay.

### Activity, thermostability and proteolysis assays of alginate lyase

Catalytic activity of alginate lyase (ID 22) was evaluated by both UV spectroscopy, using alginate as substrate at pH 7.5 and 37 °C, and DNSA method. The enzyme showed an activity of 1.52 ± 0.026 AU/min @233 nm and 0.282 ± 0.0025 g reducing sugars/L × min.

**Figure 4:**
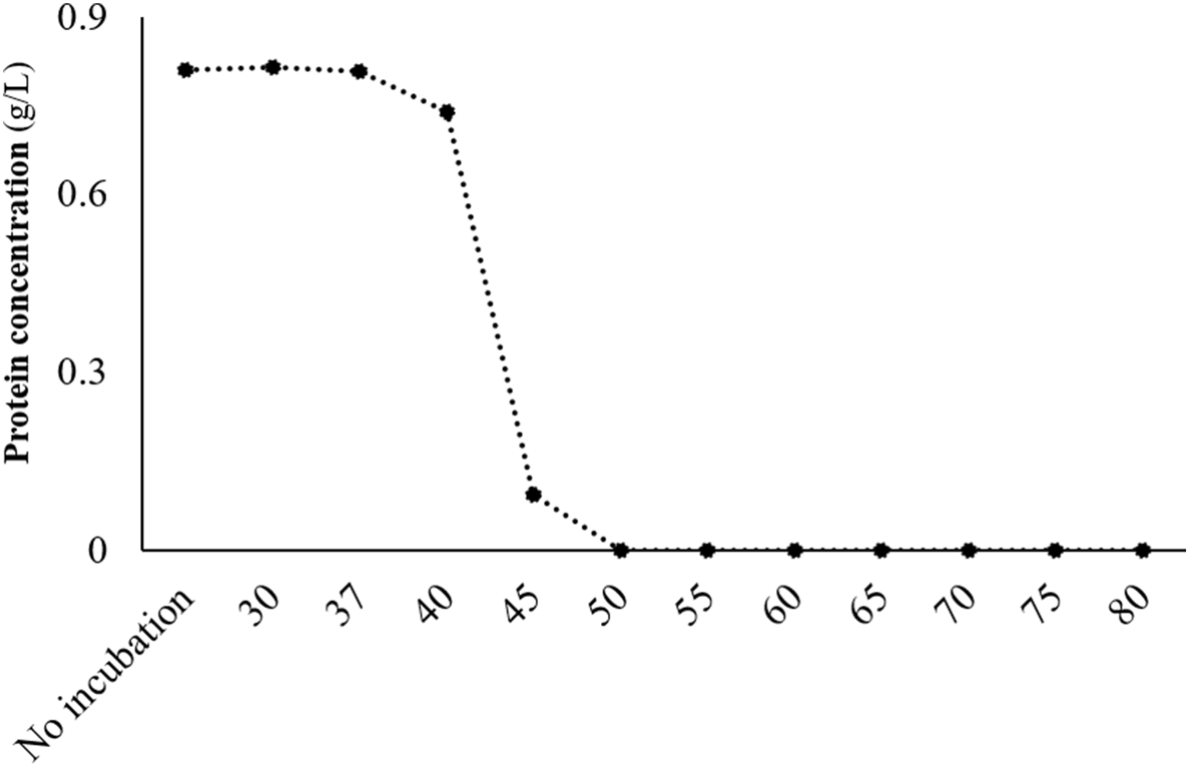
Thermostability analysis of alginate lyase (ID 22) at different temperatures (30 to 80 ºC).

**Figure 5:**
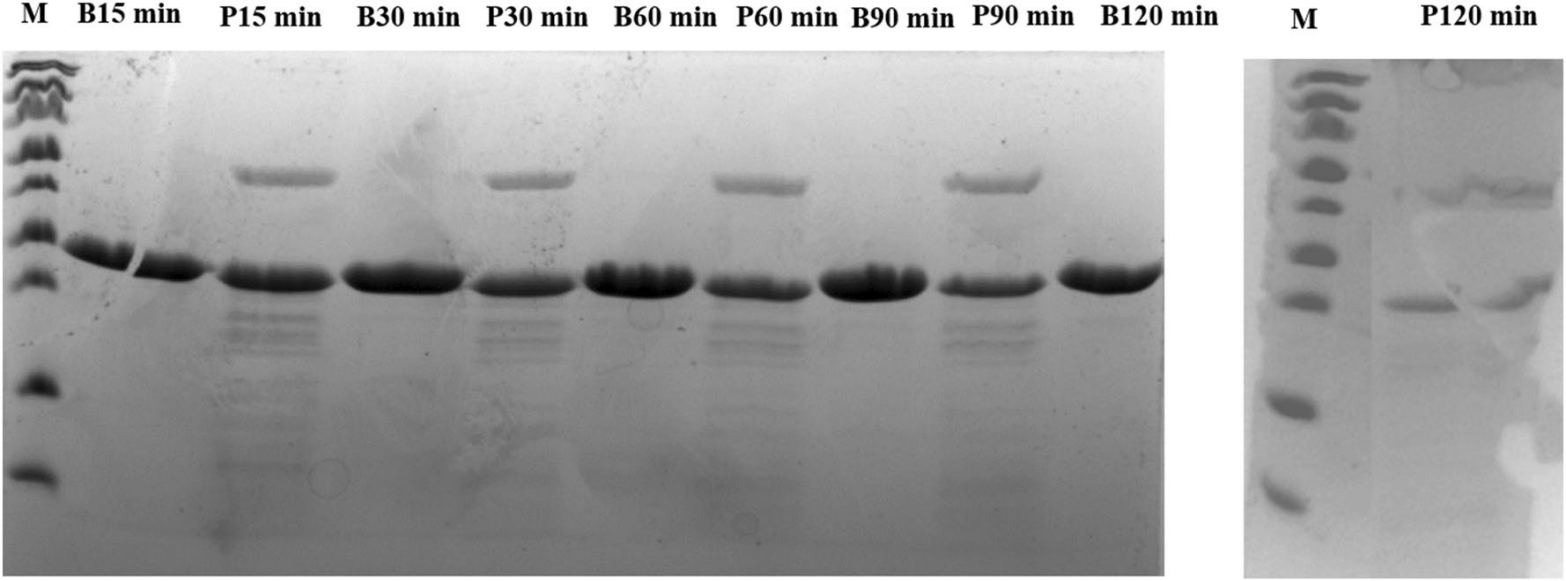
Proteolysis assay results. Electrophoresis on SDS-PAGE in 14% (w/v) acrylamide gels displaying the fragment bands of alginate lyase (ID 22) (0.83 g/L) after incubation with pancreatin (final concentration of 2.5 g/L). The gel band of 31.8 KDa corresponds to purified protein, whereas the other bands correspond to degraded protein and pancreatin. B: blank (purified undigested ID22) P: purified protein submitted to hydrolysis by pancreatin, M: protein marker.

The purified (> 90% purity) alginate lyase was tested for its thermostability and proteolysis resistance. For the thermostability assay, the intact protein was subjected to a range of temperatures (30 to 80 ºC) (Fig. 4). The enzyme maintained its stability at 37 and 40 ºC. Although significant (p < 0.001), only a small variation of protein concentration was found between these two temperatures (0.81 to 0.74 g/L, respectively). However, the stability of alginate lyase declined abruptly between 40 °C and 50 ºC, with the enzyme being completely degraded at 50 ºC. The proteolytic resistance of alginate lyase is shown in Table 3 and Fig. 5. The enzyme showed partial resistance over the entire assay time.

**Table 3:** Proteolysis resistance for alginate lyase (ID 22) at a concentration of 0.83 g/L, when subjected to the proteolytic action of pancreatin, which was incubated at a final concentration of 2.5 g/L.

ID	Time (min)				
	15	30	60	90	120
22	+	+	+	+	+

The reactions were incubated at 37 ºC, at regular intervals of 15 min for 120 min. Results are presented at periods of 15, 30, 60, 90 and 120 min of incubation. The qualitative scale of proteolysis resistance is based on SDS-PAGE gels visualisation: -, no resistant (only fragmentation bands); + , partially resistant (protein and fragmentation bands).

### Effect of alginate lyase on the release of mono- and oligosaccharides from *Laminaria digitata* cell wall

**Figure 6:**
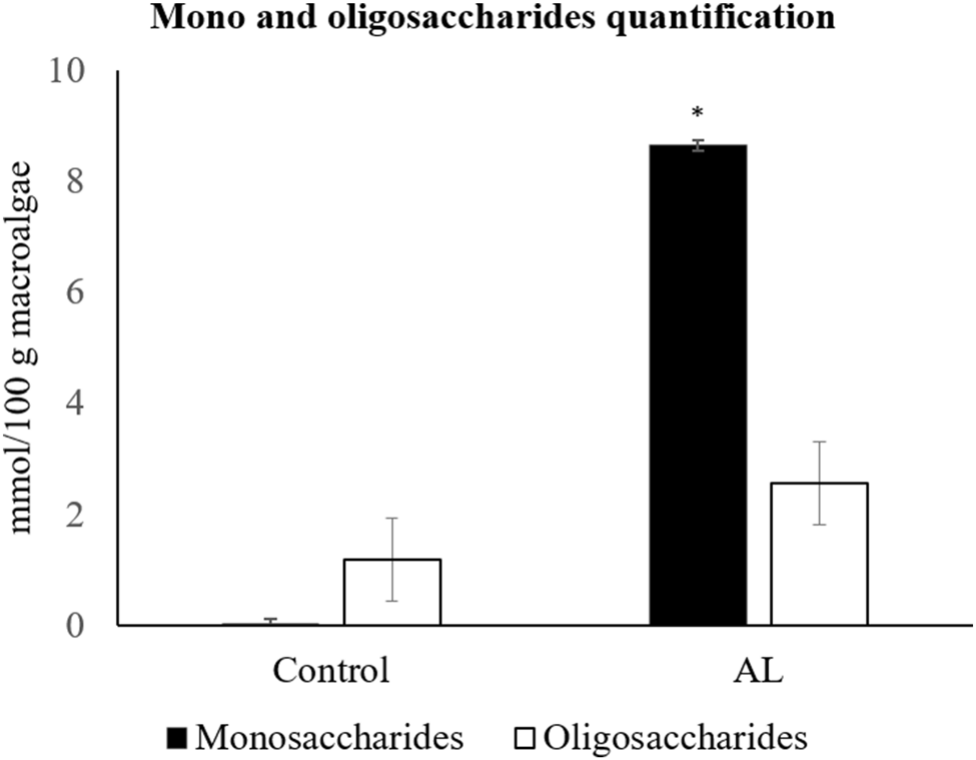
Quantification of released mono- and oligosaccharides for control assay and alginate lyase (AL; ID 22) treatment. Asterisk denotes statistical difference at *p* < 0.001.

Figure 6 shows the influence of alginate lyase treatment on the release of mono- and oligosaccharides from *L. digitata* cell wall. The composition of mono and oligosaccharides was a mixture of compounds, not individually identified due to its complexity and lack of some commercial standards. With the enzyme treatment, monosaccharide concentrations significantly increased (*p* < 0.001), from 0.02 to 8.64 mmol/100 g dried alga, in relation to the control. Although the amount of oligosaccharides did not significantly differed (*p* = 0.260) between assays, a numerical increase, from 1.19 to 2.57 mmol/100 g dried alga, was found for the alginate treatment. In addition, residual amounts of glucose (8.24 × 10^–4^ mmol/100 g dried alga) were released from *L. digitata* biomass with the alginate lyase treatment (data not shown).

### Effect of alginate lyase on the release of proteins and pigments from *Laminaria digitata* biomass

The influence of alginate lyase (ID 22) treatment on pigment and protein concentrations in the supernatant and residue fractions is presented in Table 4. These results indicate if the hydrolysis of the viscous gel-like structure formed by the polymer alginate^11^ led to the release of trapped valuable nutrients. The incubation of alga with the enzyme did not trigger (*p* > 0.100) the release of protein from *L. digitata* cells and, thus, a similar protein content was found for the enzyme and control assays (31.1 and 39.9 mg/g alga for the supernatant, and 114 and 91.7 for the residue). Additionally, no significant differences (*p* > 0.071) between assays were observed for chlorophyll, carotenoid and fucoxanthin contents in both centrifugation fractions.

**Table 4:** Content (mg/g alga) of total proteins, chlorophylls, carotenoids, fucoxanthins and fatty acids of the supernatant and residue fractions derived from the incubation of *Laminaria digitata* with control and alginate lyase (AL; ID 22) treatment.

	Supernatant				Residue			
	Control	AL	SEM	*p*-value	Control	AL	SEM	*p*-value
Total protein	39.9	31.1	5.14	0.289	91.7	114.3	8.72	0.167
Chlorophyll *a*	0.013*	0.014	0.0019	0.643	0.124**	0.119	0.0028	0.274
Chlorophyll *b*	0.015*	0.018	0.0032	0.590	0.006**	0.005	0.0028	0.874
Total chlorophylls	0.028*	0.032	0.0051	0.612	0.130**	0.124	0.0050	0.443
Total carotenoids	0.024*	0.031	0.0053	0.413	0.034**	0.029	0.0015	0.071
Total chlorophylls + Total carotenoids	0.053*	0.063	0.0083	0.418	0.164**	0.153	0.0058	0.248
Total pheophytins	0.121*	0.140	0.0253	0.630	0.552**	0.534	0.0248	0.641
Fucoxanthins	0.021*	0.021	0.0011	0.842	0.016**	0.013	0.0010	0.084
Total fatty acids	1.30^b^	4.22^a^	0.233	0.001	1.34	1.82	0.432	0.516
*Fatty acid composition (% total fatty acids)*								
12:0	0.61^a^	0.33^b^	0.032	0.004	0.58	0.56	0.040	0.798
14:0	2.24^a^	1.27^b^	0.136	0.007	3.01	3.88	0.880	0.555
15:0	0.76	0.44	0.102	0.090	0.72	0.55	0.230	0.652
16:0	35.45^a^	22.18^b^	0.432	< 0.001	36.31	31.59	1.772	0.200
16:1*c*9	0.75^b^	1.13^a^	0.082	0.029	1.54	1.80	0.240	0.521
17:0	3.32	2.50	0.354	0.179	3.71	2.47	0.901	0.433
18:0	34.21^a^	28.27^b^	1.176	0.023	25.60	23.51	2.242	0.578
18:1*c*9	10.16^b^	31.94^a^	1.388	< 0.001	13.31	18.26	2.752	0.331
18:1*c*11	1.07^b^	2.29^a^	0.230	0.020	1.14	1.46	0.339	0.579
18:2n-6	2.08	1.35	0.190	0.053	2.63	2.69	0.479	0.943
18:3n-3	0.30	0.26	0.112	0.845	1.04	1.30	0.550	0.772
20:0	1.74^a^	0.62^b^	0.104	0.002	1.48	1.33	0.149	0.534
20:1*c*11	0.21^b^	0.89^a^	0.154	0.036	0.54	1.04	0.119	0.096
20:4n-6	0.18	0.17	0.130	0.965	2.54	2.01	0.710	0.655
20:5n-3	0.34	0.24	0.248	0.776	1.90	1.66	0.468	0.752
21:0	3.15	3.09	1.667	0.980	1.38	2.48	1.029	0.527
22:0	1.59^a^	0.51^b^	0.259	0.043	1.08	1.15	0.296	0.874
22:1	1.54	0.65	0.250	0.065	1.20	1.03	0.238	0.669
Others	0.29^b^	1.86^a^	0.269	0.015	0.31	1.24	0.250	0.119
∑ SFA	83.07^a^	59.21^b^	1.801	0.001	73.87^a^	67.53^b^	0.893	0.037
∑ MUFA	13.73^b^	36.90^a^	1.442	< 0.001	17.71	23.58	2.603	0.252
∑ PUFA	2.91	2.02	0.365	0.163	8.11	7.66	2.204	0.899
∑ n-3 PUFA	0.64	0.50	0.287	0.748	2.94	2.96	1.017	0.991
∑ n-6 PUFA	2.26^a^	1.52^b^	0.172	0.038	5.17	4.70	1.188	0.807

Two mL of macroalgae suspension was incubated with alginate lyase at a final concentration of 20 mg/L. The control assay took the same amount of PBS. Incubations were done overnight at 37 ºC and 160 rpm. After incubations, supernatant and residue fractions were separated by centrifugation. Only fatty acids whose percentage was > 0.5% are presented. * Values measured in phosphate buffered saline (PBS); ** Values measured after extraction with acetone.

### Effect of alginate lyase on the release of fatty acids from *Laminaria digitata* biomass

Fatty acid profile in residue and supernatant fractions, after incubation with the alginate lyase (ID 22), was analysed to determine if the enzyme treatment led to the release of fatty acids from *L. digitata* cells to the external environment (Table 4), since these nutrients could have been trapped by the gel-forming structure of alginate.

For both supernatant and residue fractions, the percentage of fatty acids were as follows: saturated fatty acids (SFA) > MUFA > PUFA > *n*-6 PUFA > *n*-3 PUFA. In the supernatant, the amount of total fatty acids was increased (*p* = 0.001) from 1.30 to 4.22 mg/g dried alga with the alginate lyase treatment. In fact, higher percentages of total MUFA (*p* < 0.001) were found in the presence of enzyme, with a major contribution of 18:1*c*9 (*p* < 0.001) and additional contributions of 16:1*c*9, 18:1*c*11 and 20:1*c*11. In addition, the percentage of total SFA was significantly decreased (*p* = 0.001) with the alginate lyase treatment, particularly of 12:0, 14:0, 16:0, 18:0, 20:0 and 22:0, as well as the amount of total *n*-6 PUFA (*p* = 0.038). The 18:2*n*-6 fatty acid tended to decrease (*p* = 0.053) with the alginate lyase treatment, although it was found in small percentages for both assays (2.08% for control and 1.35% for the enzyme).

In the residue fraction, the alginate lyase treatment caused no significant differences (*p* > 0.096) either in the amount of total fatty acids or in the percentage of individual fatty acids, leading only to a significant decrease of total SFA (*p* = 0.037) comparatively to the control.

## Discussion

A large library of 176 CAZymes and 23 sulfatases was produced to test the hypothesis that some of these enzymes, with well-characterized biochemical characteristics (see Supplementary Table S1), could disrupt the recalcitrant cell wall of *L. digitata* with the consequent increase of nutrients availability. The production of enzymes was done in a high-throughput (HTP) platform and consisted of several steps, including gene synthesis, gene cloning, recombinant protein expression in *E. coli* cells and protein purification. These 199 enzymes were selected based on the polysaccharide matrix composition of macroalga cell wall, which comprises mainly alginate and fucose-containing sulphated polysaccharides, as well as minor amounts of cellulose, putative hemicellulose^11^ and mixed-linked β-glucans^12^. Laminarin^13–15^ is the main carbon storage of brown seaweeds. In addition, the enzyme origin was also attended in the selection, being 121 of them from marine and halophilic bacteria and 41 from thermophilic or hyperthermophilic bacteria.

An individual screening of the enzymes was performed in order to assess their ability to degrade *L. digitata* cell wall, which was evaluated by measuring the release of reducing sugars and the intensity of microscopic fluorescence. Afterwards, the 8 selected recombinant enzymes (see Table 1) were combined and tested in order to obtain a maximum disruption of *L. digitata* cell wall, and then reduced to a combination of 3 enzymes (laminarinase ID 18, alginate lyase ID 22 and α-L-fucosidase ID 46). However, the alginate lyase led to a degradation of macroalga cell wall similar to the simultaneous use of the 3 enzymes. The absence of synergistic and additive effects between enzymes indicates that, as previously reported for brown macroalgae^11^, the different polysaccharides of *L. digitata* cell wall, namely alginate and FCSFs, are not interlinked within the cell wall structure.

This enzyme (ID 22) belongs to the family of polysaccharide lyase 7 (PL7) and is produced by *S. degradans*^35^. It possesses poly-β-mannuronate (EC 4.2.2.3) and, to a lesser extent, poly-β-guluronate (EC4.2.2.11) lyase activities. The alginate lyase endolytically depolymerize alginate by β-elimination, having both alginate and oligo-alginates as its main substrates^35^. This enzyme (ID 22) showed to be resistant to proteolysis and thermostable until 40 ºC, with an abrupt decrease of thermostability between 40 and 50 ºC. These results are likely due to the tertiary structure of protein that confers both thermotolerance and an inherent proteinase resistance^37^. The instability of this enzyme at high temperatures (> 50 ºC) over an increasing period of time was already reported^35^, even though maximum catalytic activity was described as 50 ºC^35^. This aspect might be explained by the fact that *S. degradans* is a mesophilic, instead of a thermophilic, Gram-negative bacterium. However, this organism is one of the strongest marine biomass degraders, being capable of hydrolysing a great variety of polysaccharides^38^.

Furthermore, the efficiency of the recombinant mannuronate-specific alginate lyase from PL7 family (ID 22) on releasing reducing sugars (7.11 g/L) from brown macroalga cell wall was firstly demonstrated herein. In fact, only one previous study^25^ reported the recovery of reducing sugars from *L. digitata* and *S. latissima* biomass (10 and 11 g/L, respectively), through the action of a commercial mannuronate-specific alginate lyase, belonging to the PL5 family (Genbank accession numbers: SUJ15243.1 and SUJ21107.1). This enzyme was produced by *Sphingobacterium multivorum* during the enzymatic pre-treatment of algae biomass for biogas production. However, in the present study, a well-characterized endotype PL5 alginate lyase (ID 41) produced by *Sphingomonas* sp. A1 (Genbank accession number BAB03312.1), which specifically acts on poly-β-mannuronate regions (EC 4.2.2.3) and depolymerizes alginate into tri- and disaccharides^39^, was significantly (*p* < 0.050) less efficient at releasing reducing sugars (1.70 g/L) than the 3 analysed enzymes from PL7 family (average of 6.07 g/L). The release of reducing sugars with alginate lyase (ID 22) is possibly related to an increase of monosaccharides and, although not significant, of oligosaccharides released from *L. digitata* biomass^40^. Although a recombinant alginate lyase with the same catalytic site as the enzyme with ID 22 was previously shown to produce unsaturated oligoalginates (degrees of polymerization of 2 to 5) but not monosaccharides^35,40^, a mixture of unidentified monosaccharides, which was probably composed of uronic acid residues but needs further analysis, was released by the enzyme treatment (ID 22) in the present study. The fact that residual amounts of glucose (8.24 × 10^–4^ mmol/100 g dried macroalga) were obtained with the alginate lyase treatment might indicate that the breaking down of viscous-gel like matrix of alginate lyase (ID 22) released trapped laminarin from the intracellular algae compartment, which became available for a possible autohydrolysis, as previously reported^23^. Although, to date, a selective effect of alginate lyase towards carbohydrates from brown seaweed cell wall was never reported, two studies evaluated the release of glucose from brown macroalgae biomass during either bioethanol production^23^ or algae saccharification^24^. The first described a slight release of glucose from *L. digitata* biomass, by using a commercial mannuronate-specific alginate lyase, from an unknown family, produced by *Sphingobacterium spiritivorum*. The latter, similarly to the present study, reported no effect of 3 recombinant alginate lyases from PL7 family on the release of glucose from *S. latissima*.

In the present study, the decrease of cell wall fluorescence intensity (39.1%) promoted by alginate lyase (ID 22) indicates a partial alga cell wall degradation, similarly to what was observed by our research team for microalgae^27,28^. Considering that Calcofluor white preferentially binds to cellulose and, to some extent, mixed-liked 1,3–1,4-β-glucans in macroalgae cell walls rather than to acidic polysaccharides^12,33^, like alginate and FCSPs in brown algae^11^, the degradation of alginate seems to have compromised the whole integrity of the intricate macroalgae cell wall structure with a decreased dye binding by cellulose and mixed-liked glucans. The cell wall disruption was likely due to the ability of enzyme with ID 22 to degrade polymannuronate residues of alginate and consequently compromise cell wall rigidity that is controlled by the gel-forming structure alginate^11^. In fact alginate from *L.digitata* cell wall was found to be mainly composed of mannuronic acid (M) instead of guluronic acid (G) residues (M/G ratio between 1.99 and 3.0)^41^. The preferential activity of alginate lyases from PL7 family on polymannuronic acid rather than on polyguluronic acid or alginate was demonstrated in a recent study^42^ when the enzyme produced by a marine fungus *Paradendryphiella salina* was incubated with 3 different brown alga species *(Ascophyllum nodosum*, *S. latissima* and *Fucus serratus*). However, the activity of PL7 alginate lyase (ID 22) is not specific for polymannuronate as this enzyme can also act on polyguluronate residues of alginate (Kim et al., 2012). Conversely, the catalytic residues of enzyme with ID 41 (Genbank accession number: Q9KWU1) were shown to strictly bind to mannuronic acid residues^39^. This latter aspect can help to explain why PL7 alginate lyase (ID 22) was more efficient on degrading *L. digitata* cell wall than the PL5 alginate lyase (ID 41), in the present study.

Conversely to carbohydrates, alginate lyase (ID 22) did not release (hydro-) soluble proteins from *L. digitata* cells to the external environment. These results can be due to the extracellular presence of phenolic compounds (*i.e.* phlorotannins) previously cross-linked to alginates^11^, which have protein-linkage properties^43^, and hydrocolloidal anionic polysaccharides (*e.g.* oligoalginates)^44^. These compounds would limit the access and quantification of proteins, through the increased viscosity of extraction medium, a phenomenon that was indeed observed in the supernatants from alginate lyase treatment, and reported for other carbohydrases^37^. Their presence was already found to be a limiting factor of protein extraction when cellulase and xylanase acted on *P. palmata*^19,21,45^. In addition, no release of pigments from *L. digitata* cells to the extracellular medium was found with the alginate lyase treatment. The amounts of pigments in control assay residues were slightly different from the values (mg/g dried alga) previously reported for *Laminaria* sp.^46^ (0.124 *versus* 0.184 for chlorophyll *a*, 0.006 *versus* 0.014 for chlorophyll *b*, 0.034 *versus* 0.026 for total carotenoids), which was probably due to the use of different solvents for pigment extraction^46^, as well as variations on alga species and harvesting season^47^. Similarly to what was reported for microalgae^27,28^, the alginate lyase (ID 22) could not disrupt the long parallel lamellae of tree thylakoids in cytoplasmic plastids of brown seaweeds that contain the light harvesting complex with photosynthetic pigments^48^. These results are explained by the absence of activity of the enzyme with ID 22 on the lipid and protein-rich plastid membrane, since this enzyme acts specifically on alginate and oligoalginates^35^.

Enzyme with ID 22 was able to release fatty acids from alga biomass to the extracellular medium (supernatant), although without a significant effect on the algal incubation residue. To date, only one study^49^ suggested the change of fatty acid profile promoted by alginate lyases on brown seaweeds, although using a alginate lyase from PL5 family acting on *Undaria pinnatifida* and with no statistical analysis of data. Thus, the present study is the first that show the significant effect of a recombinant alginate lyase (PL7) on fatty acid profile of a brown macroalga (*L. digitata*). It was observed an increase of total MUFA, such as 16:1*c*9, 18:1*c*9, 18:1*c*11 and 20:1*c*11 fatty acids, and a concomitant decrease of total SFA, including the major 16:0 and 18:0 fatty acids, released to the supernatant. In fact, oleic acid (18:1*c*9) was increased by threefold with the alginate lyase treatment. These results might be explained by the release of phlorotannins to the extraction medium by the action of alginate lyase, as previously reported^11^. In fact, tannins were previously shown to inhibit the complete biohydrogenation of C18 fatty acids in animals^50^. The latter aspects need to be further exploited due to the benefits that increasing the release of MUFA, such as 18:1*c*9, in detriment of SFA, have to human health, particularly on preventing cardiovascular diseases^7^.

## Conclusion

The results obtained in the present study indicate that the sole use of an alginate lyase from PL7 family, under physiological conditions, can lead to a partial degradation of *L. digitata* cell wall. The disruption of macroalga cell wall would allow the release of trapped bioactive compounds with important value for biotechnological and feed industries. The high nutritional value of these compounds may stimulate the use of exogenous enzymes, as novel biocatalysts, to supplement diets containing *L. digitata* for monogastric animals. Further work is currently in progress in our research laboratories to assess the effectiveness of using this alginate lyase as a supplement for monogastric diets with high incorporation levels (10–15% of diet weight) of *L. digitata*.

## Methods

### Macroalga production

The low heat-dried brown macroalgae *L. digitata* was obtained from Algolesko Company (Plobannalec-Lesconil, Brittany, France), where it was cultivated in seawater offshore ponds and biologically certified by Ecocert. Before it was used for the in vitro incubations, the macroalgae was ground in a knife mill (Grindomix GM 200, Retsch Gmbh, Germany), sieved through a woven wire mesh sieve with a diameter of 63 µm (Retsch Gmbh, Germany) and stored at -20 ºC.

### High-throughput gene synthesis, cloning and protein expression/purification of recombinant enzymes

One-hundred and seventy-six CAZymes with high potential for degradation of macroalgae cell wall were selected from a diverse repertoire, including glycoside hydrolases (GH), pectate lyases (PL) and carbohydrate esterases (CE). In addition, 23 sulfatases were selected for screening, as they are also likely involved in the degradation of sulphated polysaccharides from macroalgae cell walls^51^.

The generation of 199 recombinant plasmids, as well as the expression and purification of the corresponding enzymes, followed the procedures described in previous studies^27,28^. One-hundred and sixty-six coding genes for the selected enzymes were synthesised in vitro using NZYGene Synthesis kit (Nzytech, Portugal), whereas the other 33 coding genes were synthesised by Twist Bioscience (San Francisco, CA, USA). The sequence of each enzyme is presented in Supplementary Table S1. After optimisation of synthetic genes for cloning and subsequent expression in *Escherichia coli*, 166 genes were directly cloned into the bacterial expression vector pHTP1 (Nzytech, Portugal) using NZYEasy Cloning & Expression kit I (Nzytech, Portugal), whereas 33 genes were cloned in pET-29b( +) (Twist Bioscience, San Francisco, CA, USA). The generated recombinant plasmids were subjected to inducible T7 promoter control, while encoding the 199 enzymes fused to an N-terminal His_6_-tag that facilitates purification through Immobilised Metal Affinity Chromatography (IMAC). All recombinant plasmids were sequenced to ensure that no mutations accumulated during gene synthesis and were used to transform *E. coli* BL21 (DE3) cells in 24 deep-well plates, followed by protein production, cell harvesting and protein purification. An IMAC high-throughput method based on an automated protocol that allows the purification of 96 proteins simultaneously was performed, as previously reported^52^ but with some modifications. Briefly, 1 mL of crude cell lysates were incubated with 200 μL of 25% Ni^2+^ Sepharose 6 Fast flow resin (GE Healthcare, IL, USA) and transferred into 96-well filter plates (Macherey–Nagel, Duren, Germany)^52^. The wells were washed twice with 1 mL of 10 mM Imidazole buffer (10 mM Imidazole, 50 mM NaHepes, 1 M NaCl, 5 mM CaCl_2_, at pH = 7.5) and once with 1 mL of 35 mM Imidazole buffer (35 mM Imidazole, 50 mM NaHepes, 1 M NaCl, 5 mM CaCl_2_, at pH = 7.5). Then, the proteins were eluted with 300 µL of 300 mM Imidazole buffer (300 mM Imidazole, 50 mM NaHepes, 1 M NaCl, 5 mM CaCl_2_, at pH = 7.5). All steps of protein purification from cell-free extracts were automated on a Tecan robot (Tecan, Switzerland), containing a vacuum manifold. Homogeneity of purified proteins and molecular mass of recombinant enzymes were determined by SDS-PAGE in 14% (w/v) acrylamide gels and compared to a low molecular weight (LMW) protein marker (18.5 to 96 KDa) (Nzytech, Portugal). The gel images were acquired with BioRad ChemiDoc XRS imaging system (Bio-Rad, Hercules, CA, USA). Protein concentration of enzyme stock solutions varied between 0.13–26.7 g/L, as determined spectrophotometrically on NanoDrop 2000/2000c (NanoDrop Technologies; Thermo Fisher Scientific, Inc., Pittsburgh, PA, USA), and the level of protein expression was determined accordingly (see Supplementary Table S1).

### Preparation of macroalga cell suspension

The preparation of *L. digitata* suspension at 20 g/L in PBS solution, including a pre-wash step, centrifugation and algae re-suspension, was done using the procedure previously described for microalgae^27^.

### Enzymatic cell wall disruption

The cell wall disruption assay was performed in triplicate and with an enzyme concentration of 20 µg/ mL of incubation volume, as previously reported^27^, but with the following changes: the incubation of the 24 well microplate (VWR Chemicals, West Chester, PA, USA) containing macroalgae suspension and alginate lyase was performed overnight at 160 rpm. Then, the microplate was centrifuged for 30 min at 3210 g and the supernatants and pellets were recovered. To precipitate and remove the enzymes, the supernatant for DNSA and HPLC analyses was boiled for 5 min, centrifuged for 5 min at 10,000 g and the supernatant recovered.

### Reducing sugars measurement

To quantify the released reducing sugars, the 3,5-dinitrosalicylic acid (DNSA) method^53^ was used as previously described for microalgae^27^, with the modification that 0.5 mL of glucose solutions or supernatants were mixed with 0.5 mL of DNSA reagent.

### Fluorescence microscopic observations

The pellets from the enzyme cell wall disruption assay, were re-suspended in 0.5 mL of PBS solution for the initial screening or 1 mL of PBS for the assays with the most-active selected enzyme. The suspension was mixed by pipetting to ensure that the algae pellets were suspended evenly. The re-suspended macroalgae biomass concentration was 40 mg/mL. The fluorochrome Calcofluor White (Sigma-Aldrich, St. Louis, Mo, USA), that binds to the cell wall^12,33,54^, was added at 10 µL to the suspensions on adhesion slides (SuperFrost Plus Menzel Gläser, Thermo Scientific, Braunschweig, Germany), followed by a solution of 10% KOH (VWR Chemicals, West Chester, PA, USA), in a proportion of 1:1:1. The fluorescence microscopic procedures were done as previously reported^27^. Cells were observed with an epifluorescence microscope and images were captured with a Leica DFC-340FX (Leica, Wetzlar, Germany) camera system, in order to determine the fluorescence intensity, expressed as arbitrary units, by using the Image J software (NIH, Bethesda, MA, USA).

### Determination of catalytic activity of alginate lyase

The catalytic activity of alginate lyase was analysed by two different methods: UV spectroscopy and determination of reducing sugars. The UV spectroscopy analysis followed the procedures described in a previous report^55^, with some modifications. Briefly, a mixture of 1 mL of PBS solution containing 1% NaCl (pH = 7.5), 0.5 mL of alginic acid from brown algae (Sigma-Aldrich, Darmstadt, Germany) and 5 µl of alginate lyase (ID 22) at 4.37 mg/mL were mixed in a quartz cuvette. The alginate was previously dissolved in a PBS solution at a concentration of 0.3%. The increase in absorbance at λmax 233 nm was continuously recorded during 1 h in an UV/Vis Spectrophotometer (Pharmacia LKB Ultrospec III spectrophotometer, Gemini, Apeldoorn, Netherlands), at 37 °C, to verify linearity. The enzyme activity was maximum during 2 min. The maximum activity was reported in absorbance units (AU) defined as the increase in absorbance units per minute. The determination of reducing sugars was done after stopping the enzyme reaction by using the DNSA reagent with subsequent heating of the samples, following the procedures previously described for the measurement of reducing sugars^27^.

### Thermostability and proteolysis experiments

The alginate lyase was biochemically characterized, specifically for thermostability and proteolysis resistance. The recombinant enzyme was purified through IMAC using gravity flow columns (His GraviTrap™, GE Healthcare, IL, USA), according to a previously described procedure^29,30^. The protein concentration was adjusted at 0.83 g/L for both assays. The thermostability analysis was performed as previously reported^27^. The protein concentration in the recovered supernatant was quantified in triplicate using a NanoDrop 2000/2000c (NanoDrop Technologies; Thermo Fisher Scientific, Inc., Pittsburgh, PA, USA), and the results were validated through visualization of 14% SDS-PAGE gels, showing the intensity of the bands present in the supernatants. The gel images were acquired with BioRad ChemiDoc XRS imaging system (Bio-Rad, Hercules, CA, USA). The proteolysis resistance analysis was performed as already described^27^. The alginate lyase was incubated with porcine pancreatin (VWR Chemicals, West Chester, PA, USA) or PBS solution and, afterwards, the samples were analysed by 14% SDS-PAGE and compared to a low molecular weight (LMW) protein marker (18.5 to 96 KDa) (Nzytech, Portugal) (Fig. 5). The resultant images were acquired with BioRad ChemiDoc XRS imaging system (Bio-Rad) and proteolysis was confirmed by visualizing fragments with different molecular weights.

### Determination of mono- and oligosaccharides

The profile of mono- and oligosaccharides from the supernatants derived from incubation of *L. digitata* with control and alginate assays was analysed and quantified by high performance liquid chromatography (HPLC). For the analysis of monosaccharide profile, the chromatogram data was compared with individual monosaccharides standards composed by arabinose, galactose, glucose, mannose plus xylose. Additional monosaccharides, including ribose, fructose, rhamnose, fucose, as well as its derivatives, such as galactosamine, glucosamine, sorbitol and glucoronic acid were also used for comparison. For the analysis of oligosaccharide profile, the chromatogram data was compared with a mixture of cellulose-derived oligosaccharides. The procedures followed a previously developed protocol^27^. The quantification of total oligosaccharides was based on a standard curve, using a range of concentrations from 0.025 to 0.60 mM of glucose. The results were expressed as equivalent moles of released glucose *per* 100 g of macroalga.

### Determination of protein content

After *L. digitata* suspension and incubation with control and alginate assays, the N content in lyophilised supernatant and residue fractions was quantified by the Kjeldahl method (984.13)^56^, assuming that no nitrogen from the media interfere with the assay. The crude protein was calculated as N × 4.92^57^.

### Pigment analysis

The content of chlorophyll *a*, chlorophyll *b*, total carotenoids and pheophytins were quantified in supernatant and residue fractions from *L. digitata* suspension, after control and alginate assays, as described by Hynstova et al.^58^, with slight modifications previously reported^27^, except that total carotenoids also included the amount of fucoxanthins. The fucoxanthin content was quantified in the same way as the other pigments, but using the following formula described in a recent study^59^:

C_fuc_ = 6.39 × A445 − 5.18 × A663, where C_fuc_ is the concentration of fucoxanthin (mg/ml), A445 is the absorbance at λmax 445 nm and A663 is the absorbance at λmax 663 nm.

### Determination of fatty acid composition

Fatty acids from the lyophilised supernatants and pellets of *L. digitata* after control and alginate assays were extracted as already described for microalgae^27^. Fatty acids were esterified to methyl esters (FAME) by acidic catalysis based on the procedure described in a previous report^4^, but using 5 ml of acetylchloride-methanol solution (1.25 M Sigma-Aldrich, St. Louis, Mo, USA) for up to 24.3 mg of sample. The analysis of FAME was done following procedures previously reported^27^, except for the quantification of total FAME, that was carried out using nonadecanoic acid (19:0) as internal standard. Each fatty acid was expressed as a percentage of the sum of identified fatty acids (% total fatty acids). The fatty acid present in a percentage inferior to 0.5% were included as others in Table 3.

### Statistical analysis

Data were analysed using the Generalised Linear Mixed (GLM) model of the SAS software package (version 9.4; SAS Institute Inc., Cary, NC, USA), except data from the thermostability experiment, which were analysed using the MIXED procedure of SAS. Normality was checked using Shapiro–Wilk test. All experiments were conducted in triplicate, except for the initial screening where experiments were conducted in duplicate. The error bars on figures indicate the standard error of the mean (SEM). Results are presented as mean and SEM, and were considered significantly different when *p* < 0.05.

## Supplementary Information

Supplementary Information 1.

Supplementary Information 2.

Supplementary Information 3.

## Data Availability

All data generated during this study are included in this published article. The datasets generated during the current study are available from the corresponding author on demand.
