# Supplementary material for: An individual alginate lyase is effective in the disruption of Laminaria digitata recalcitrant cell wall
Source: Sci Rep. 2021 May 6;11:9706. doi: 10.1038/s41598-021-89278-1 (PMC8102539; doi:10.1038/s41598-021-89278-1)

**An individual alginate lyase is effective in the disruption of *Laminaria digitata* recalcitrant cell wall**

Mónica Costa, Luís Pio, Pedro Bule, Vânia Cardoso, Cristina M. Alfaia, Diogo Coelho, Joana Brás, Carlos M. G. A. Fontes, José A. M. Prates

**Supplementary Fig. S1**. SDS-PAGE in 14% (w/v) acrylamide gels displaying the fragment bands obtained by electrophoresis of some total insoluble protein extract (T) and enriched soluble (S) fraction of recombinant proteins (the numbers correspond to identification number, ID). The gel bands corresponding to target proteins are highlighted within white rectangles. M: protein marker.

M T69 S**69** T77 S**77** T85 S**85** T93 S**93** T6 S**6** T14 S**14** T22 S**22**


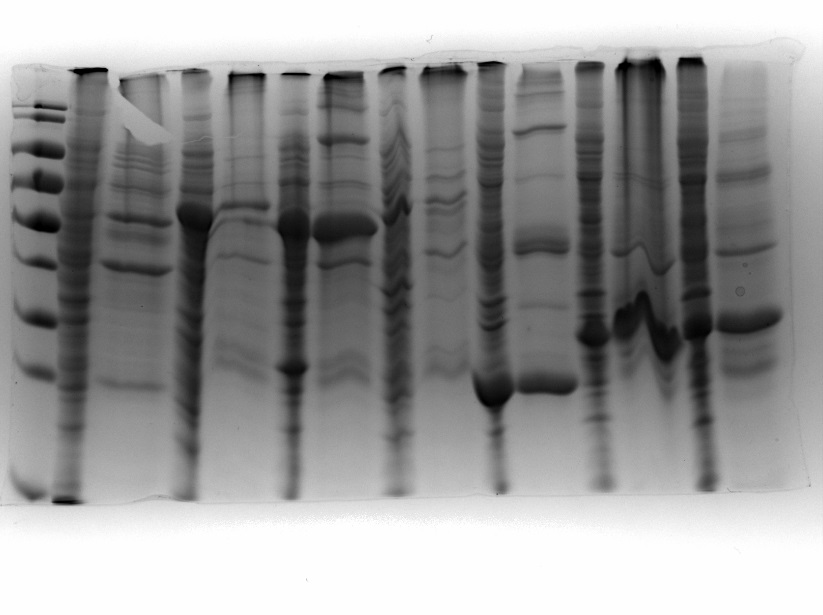


S**30** T38 S**38** M T46 S**46** T54 S**54** T62 S**62** T70 S**70** T78 S**78**


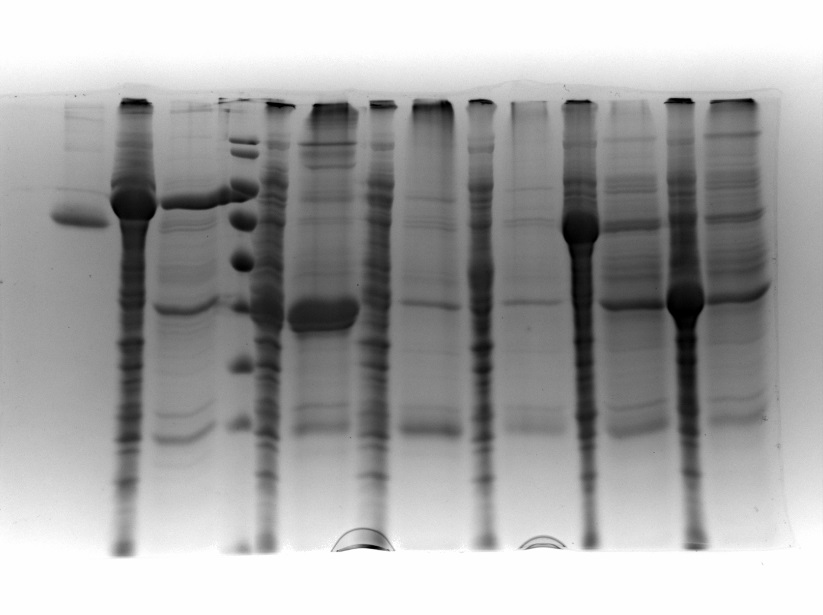

Supplement: Supplementary file 1 — Supplementary Information 1. [file 41598_2021_89278_MOESM1_ESM.docx]
